# Serum metabolomic alterations in Beagle dogs experimentally infected with *Toxocara canis*

**DOI:** 10.1186/s13071-019-3703-5

**Published:** 2019-09-11

**Authors:** Wen-Bin Zheng, Yang Zou, Hany M. Elsheikha, Guo-Hua Liu, Min-Hua Hu, Shui-Lian Wang, Xing-Quan Zhu

**Affiliations:** 1 Hunan Provincial Key Laboratory of Protein Engineering in Animal Vaccines, Hunan Engineering Technology Research Center of Veterinary Drugs, College of Veterinary Medicine, Hunan Agricultural University, Changsha, 410128 Hunan People’s Republic of China; 2 State Key Laboratory of Veterinary Etiological Biology, Key Laboratory of Veterinary Parasitology of Gansu Province, Lanzhou Veterinary Research Institute, Chinese Academy of Agricultural Sciences, Lanzhou, 730046 Gansu People’s Republic of China; 3 Faculty of Medicine and Health Sciences, School of Veterinary Medicine and Science, University of Nottingham, Sutton Bonington Campus, Loughborough, LE12 5RD UK; 4 National Seed Center of Experimental Dogs, Guangzhou General Pharmaceutical Research Institute Co. Ltd, Guangzhou, 510240 Guangdong People’s Republic of China

**Keywords:** *Toxocara canis*, Toxocariasis, Beagle dog, Serum, Metabolomic, LC-MS/MS

## Abstract

**Background:**

*Toxocara canis*, a globally distributed roundworm, can cause debilitating disease in dogs and humans; however, little is known about the metabolomic response of the hosts to *T. canis* infection. There is an increasing need to understand the metabolic mechanisms underlying the pathogenesis of *T. canis* infection in dogs. Here, we examined the metabolomic changes in Beagle dogsʼ serum following *T. canis* infection using LC-MS/MS.

**Results:**

The metabolic profiles of Beagle dogsʼ serum were determined at 12 h, 24 h, 10 d and 36 d after oral infection with 300 infectious *T. canis* eggs by LC-MS/MS. We tested whether the *T. canis*-associated differentially abundant metabolites could distinguish the serum of infected dogs from controls, as measured by the area under the receiver operating characteristic (ROC) curve (AUC). The differentially expressed metabolites were further evaluated by principal components analysis and pathway enrichment analysis. A total of 5756 and 5299 ions were detected in ESI+ and ESI− mode, respectively. ROC curve analysis revealed nine and five metabolite markers, at 12 hpi and 24 hpi to 36 dpi, respectively, with potential diagnostic value for toxocariasis. The levels of taurocholate, estradiol, prostaglandins and leukotriene were significantly changed. Primary bile acid biosynthesis pathway, steroid hormone biosynthesis pathway and biosynthesis of unsaturated fatty acids pathway were significantly altered by *T. canis* infection.

**Conclusions:**

These findings show that *T. canis* infection can induce several changes in the dog serum metabolome and that the metabolic signature associated with *T. canis* infection in dogs has potential for toxocariasis diagnosis.

## Background

The canine zoonotic roundworm *Toxocara canis* is widely distributed all over the world, with a predilection for people in impoverished communities [1, 2]. *Toxocara canis* is the causative agent of a serious or even a debilitating disease, toxocariasis, whose clinical course is remarkably heterogeneous. Infected individuals can develop ocular larva migrans (OLM), visceral larva migrans (VLM), neurotoxocariasis (NT) or covert toxocariasis (CT) [2]. Toxocariasis is one of the five neglected parasitic infections prioritized by the CDC for public health action [3] (https://www.cdc.gov/parasites/npi/). Without leveraging cutting-edge scientific approaches to counter this parasite, toxocariasis is expected to be amongst the major global parasitic infections by the year 2050 [4].

To complete its complex life-cycle, *T. canis* employs various pathways to adapt inside the vertebrate host and the eggs have a remarkable ability to withstand adverse environmental conditions, which makes effective control of this parasite a difficult task [5]. Emerging high-throughput ‘omics’ technologies, such as genomics, transcriptomics, proteomics and metabolomics, have been employed to shed light on the pathogenesis of parasitic diseases [6–9]. Additionally, mass spectrometry has been used to characterize somatic proteins and excretory–secretory products of *T. canis* [10, 11]. Although, a considerable amount of information pertaining to genetic framework of *T. canis* is available [12], there is a dearth of information on the molecular mechanisms that underpin the pathophysiology of toxocariasis. Furthermore, the metabolic signature specific for *T. canis* infection in dogs is still unknown.

Metabolomics can be efficiently used to investigate global metabolic profiles since it provides a means to quantify and discriminate metabolites that are associated with a pathophysiological process [13, 14]. In this study, we investigated the differences in the serum metabolic profiles of Beagle dogs with *T. canis* infection at different stages, compared with healthy uninfected dogs using liquid chromatography–tandem mass spectrometry (LC-MS/MS)-based metabolomics approach. Our data provided another layer of information about the regulatory metabolic constitution that underpins the adaptation of toxocariasis during different stages of infection in the canine host.

## Methods

### Embryonated eggs

Unembryonated eggs of *T. canis* were collected from the uteri of fertile *T. canis* females. The unembryonated eggs were incubated on filter papers with 0.5% formalin solution under 28 °C with 85–95% relative humidity for 28 days. The embryonated eggs were collected from the filter papers and filtered with 200 mesh screens, then stored in 1% formalin solution at 4 °C.

### Animals and experimental infection

Fifty-one SPF Beagle dogs aged 6 to 7 weeks-old were purchased from the National Seed Center of Experimental Dogs in accordance with GB 14922.2-2011 “Laboratory animal-microbiological standards and monitoring”. All puppies were raised in the Good Laboratory Practice (GLP) large animal laboratory in the National Seed Center of Experimental Dogs without vaccinating and feeding any medication. These puppies were divided into 4 groups: 12 h group; 24 h group; 10 d group; and 36 d group (Table 1). Puppies from the same litters were split equally between infection group and control group to reduce background differences. Stool examination by the sugar water floating method and routine blood examination using automatic blood analyzer (XT2000 iv; Sysmex, Kobe, Japan) were performed to ensure all puppies were intestinal parasitic infection free. Anti-*T. canis* IgG antibody was examined by indirect ELISA using larval ES antigen to ensure all puppies were negative of *T. canis* infection as previously described [15]. Puppies of the infected groups were infected orally with 300 embryonated *T. canis* eggs, and puppies of the control groups were mock-inoculated with same amount saline after one week of the environmental adaptation in animal laboratory.

**Table 1:** Summary of the differential ions at different infection stages

Group	Number	Ion mode	Difference in ion number	Up^a^	Up^a^ (MS)	Up^a^ (MS_2_)	Down^a^	Down^a^ (MS)	Down^a^ (MS_2_)	MS	MS_2_
A12hT *vs* A12hC (12 hpi)	7 *vs* 7	ESI(+)	59	31	19	10	28	19	9	38	19
		ESI(−)	81	37	22	8	44	23	9	45	17
B24hT *vs* B24hC (24 hpi)	6 *vs* 6	ESI(+)	417	330	191	145	87	55	30	246	175
		ESI(−)	91	34	17	11	57	19	12	36	23
D10dT *vs* D10dC (10 dpi)	7 *vs* 6	ESI(+)	500	137	91	55	363	265	181	356	236
		ESI(−)	814	271	150	87	543	358	227	508	314
F36dT *vs* F36dC (36 dpi)	6 *vs* 6	ESI(+)	285	138	80	60	147	98	48	178	108
		ESI(−)	268	72	30	16	196	88	40	118	56

^a^Up and down indicate upregulated and downregulated differential ion number, respectively

*Abbreviations*: MS, first mass spectrometry; MS_2_, secondary mass spectrometry

### Preparation of the blood samples

A blood sample from each puppy was collected only once, either at 12 h post-infection (hpi), 24 hpi, 10 days post-infection (dpi) or 36 dpi from the jugular vein, depending on the assigned group. One part of the blood was collected aseptically into tubes with EDTA-K_2_ for routine blood examination and another part of the blood was collected into tubes without anticoagulant for harvesting serum. The blood was placed at ambient temperature for 1 h, and then the serum was obtained by centrifugation at 10,000×*g* for 10 min at 4 °C. Serum was divided into 1.5 ml tubes and stored at − 80 °C for non-targeted LC-MS/MS analysis.

### Recovery of *T. canis* and eggs at different infection stages

In order to further confirm the infection of *T. canis* and minimize the number of animals used, only puppies from the 12 h, 24 h and 36 d groups were humanely killed by KCl under a general anesthetic treatment using Zoletil 50 (Virbac, Nice, France), according to the migration pathways of *T. canis*. Puppies in the 10 d group were fed albendazole after the experiment, according to veterinary guidance. The liver was shredded to recover larvae by the modified Baermann funnel method for 12 h at 37 °C with 1% mycillin [16]. Fecal eggs of 36 d group dogs were daily examined from 30 to 36 dpi to determine the status of *T. canis* infection.

### Extraction of genomic DNA from *T. canis*

Genomic DNA was extracted from the embryonated eggs used to infect puppies, and larvae and adults of *T. canis* recovered from infected puppies using a commercial DNA extraction kit according to the manufacturer’s instructions (TianGen™, Beijing, China). DNA samples were used to amplify the ITS region of *T. canis* by PCR, as previously described [17]. Positive PCR products were submitted to Sangon Biotech (Shanghai, China) for sequencing. Obtained sequences were aligned with reference sequences available in GenBank using the Basic Local Alignment Search Tool (BLAST).

### LC-MS/MS-based metabolomics analysis

Liquid chromatography was performed using the 2777C ultra-performance liquid chromatography (UPLC) system (Waters, Manchester, UK) as previously described [18]. A high-resolution tandem mass spectrometer Xevo G2 XS QTOF (Waters) was used to detect metabolites eluted from the column. The Q-TOF was operated in both positive and negative ion modes. For positive ion mode, the capillary and sampling cone voltages were set at 3.0 kV and 40.0 V, respectively. For negative ion mode, the capillary and sampling cone voltages were set at 2.0 kV and 40.0 V, respectively. The mass spectrometry data were acquired by Centroid MSE mode. The TOF mass range ranged from 50 to 1200 Da and the scan time was 0.2 s. For the MS/MS detection, all precursors were fragmented using 20–40 eV, and the scan time was 0.2 s. During the acquisition, the LE signal was acquired every 3 s to calibrate the mass accuracy. Furthermore, a quality control (QC) sample (pooled aliquots from all analyzed samples) was analyzed every 6 samples and used to evaluate the consistency of the LC-MS during the whole acquisition.

### Data analysis

Peak extraction was implemented by commercial software Progenesis QI v.2.2, utilizing raw data (all samples and QC samples), including peak alignment, peak extraction, normalization, deconvolution and compound identification. The data were adjusted by quality control-based robust LOESS signal correction (QC-RLSC) [19]. Then, low-quality ions whose relative standard deviation (RSD) was > 30% compared to QC samples were filtered out. Metabolites were identified based on databases of Human Metabolome Database (HMDB; http://www.hmdb.ca) and Kyoto Encyclopedia of Genes and Genomes (KEGG; http://www.genome.jp/kegg). Principal components analysis (PCA) and partial least squares discriminant analysis (PLS-DA) were performed to discriminate infected puppies and control puppies. In this study, the VIP threshold of the first two principal components of the multivariate PLS-DA model combined with univariate analysis of fold-change (FC) and *P*-values were used to identify the differentially abundant metabolites, as follows: VIP ≥ 1 and FC ≥ 1.2 or ≤ 0.8333 and *P* < 0.05. The areas under the receiver operating characteristic (ROC) curves (AUC) of all identified metabolites were analyzed to predict potential biomarkers using MetaboAnalyst v.4.0 [20] (https://www.metaboanalyst.ca). Cluster analysis was performed to reveal the distinct metabolomic signature between infected and control puppies using the *heatmap* package in R based on the differentially abundant metabolites which were converted to log2 scale. Analysis of metabolic pathways was performed based on the KEGG database to explore the main biochemical pathways involved in *T. canis* infection. The numbers of eosinophils were analyzed by *t*-test using GraphPad Prism v.5.

## Results

### Confirmation of *T. canis* infection in Beagle dogs

**Fig. 1:**
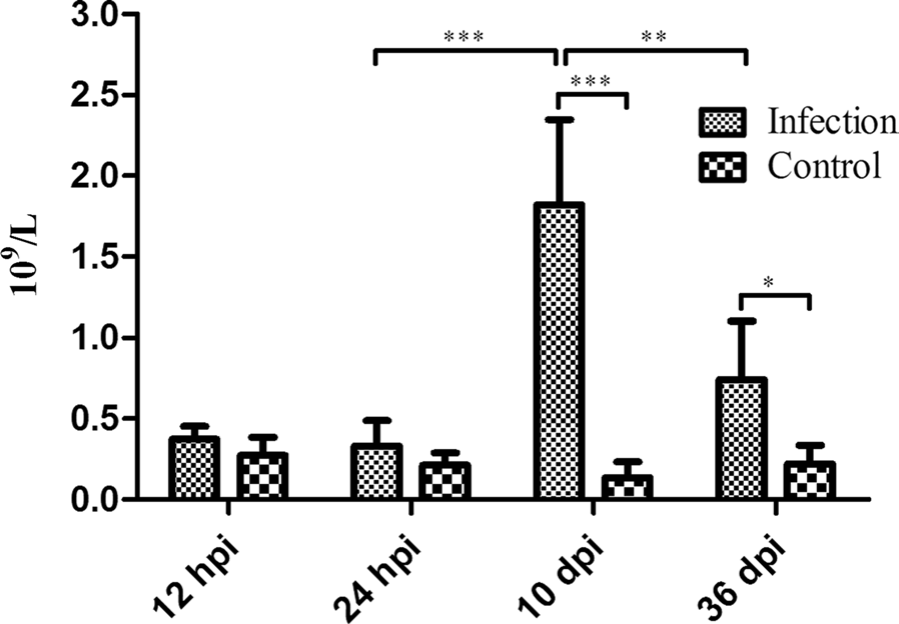
The number of eosinophils in the infected and control groups at the four indicated infection stages in the blood of Beagle dogs infected with 300 *T. canis* embryonated eggs were analyzed by t-test using GraphPad Prism v.5. [24 h infection group *vs* 10 d infection group: *t*_(11)_ = 6.64, *P* < 0.001; 10 d infection group *vs* 10 d control group: *t*_(11)_ = 7.67, *P* < 0.001; 10 d infection group *vs* 36 d infection group: *t*_(11)_ = 4.22, *P* < 0.01; 36 d infection group *vs* 36 d control group: *t*_(10)_ = 3.37, *P* < 0.05]. *P < 0.05, **P < 0.01, ***P < 0.001

Puppies used in the study were *T. canis*-free based on the absence of eggs, normal level of blood eosinophils and negative anti-*T. canis* IgG antibody. Eosinophilia was observed in infected puppies at all infection stages. The results were significantly different between infected and control animals at 10 and 36 dpi (Fig. 1). As expected, there were no significant differences between all four control groups (one-way ANOVA, *F*_(3, 21)_ = 1.95, *P* = 0.1519). Anti-*T. canis* IgG antibodies were detected in animals at 10 and 36 dpi. At 12 hpi, *T. canis* larvae were found in the livers of 2 puppies; at 24 hpi *T. canis* larvae were found in the livers of all puppies; at 36 dpi, *T. canis* were found in the livers of 2 puppies and in the small intestines of all puppies (Additional file 1: Table S1); and no *T. canis* were found in all control groups. No obvious clinical symptoms were observed in infected puppies, but the infected and control puppies could be still distinguished by the difference in the number of blood eosinophils, the level of anti-*T. canis* IgG antibody or the metabolic signatures. These differences reached a maximum level at 10 dpi, and then began to decline, indicating that the severity of toxocariasis was correlated with the distribution or growth stage of *T. canis*. In this study, the embryonated eggs used to infect puppies, and tissue larvae and adults of *T. canis* recovered from infected puppies were also subjected to PCR, and their sequences had 99% similarity to a published sequences of *T. canis* from Sichuan Province, China (GenBank: JF837169.1).

### Serum metabolic profiles

**Fig. 2:**
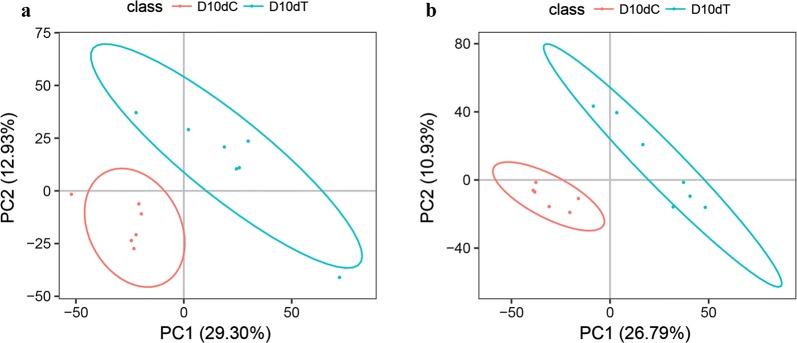
Partial least squares discriminant analysis (PLS-DA) score scatter plots of ions at 10 dpi in Beagle dogs infected with 300 *T. canis* embryonated eggs. **a** PLS-DA score plots of the control group (C) and infection group (T) at 10 dpi in ESI+ mode. **b** PLS-DA score plots of the control group (C) and infection group (T) at 10 dpi in ESI− mode

We characterized serum metabolomic changes in *T. canis*-infected (*n* = 26) *versus* uninfected (*n* = 25) puppies using LC-MS/MS. A total of 8052 and 7843 ions were identified in ESI+ and ESI− mode, respectively. After filtering low-quality ions which had RSD > 30% in QC samples, 5756 and 5299 ions were retained in ESI+ or ESI− mode, respectively. The PCA scores plots of the QC samples, infected groups and control groups are shown in Additional file 2: Figure S1, where QC samples were clustered closely, indicating the good reproducibility of the chromatographic separation of the metabolomic analysis in this study. However, the infected groups and the control groups were not separated by PCA scores plots. PLS-DA analysis showed clear separation between infected and control groups (Fig. 2 and Additional file 3: Figure S2).

### Metabolome alterations at different infection stages

**Fig. 3:**
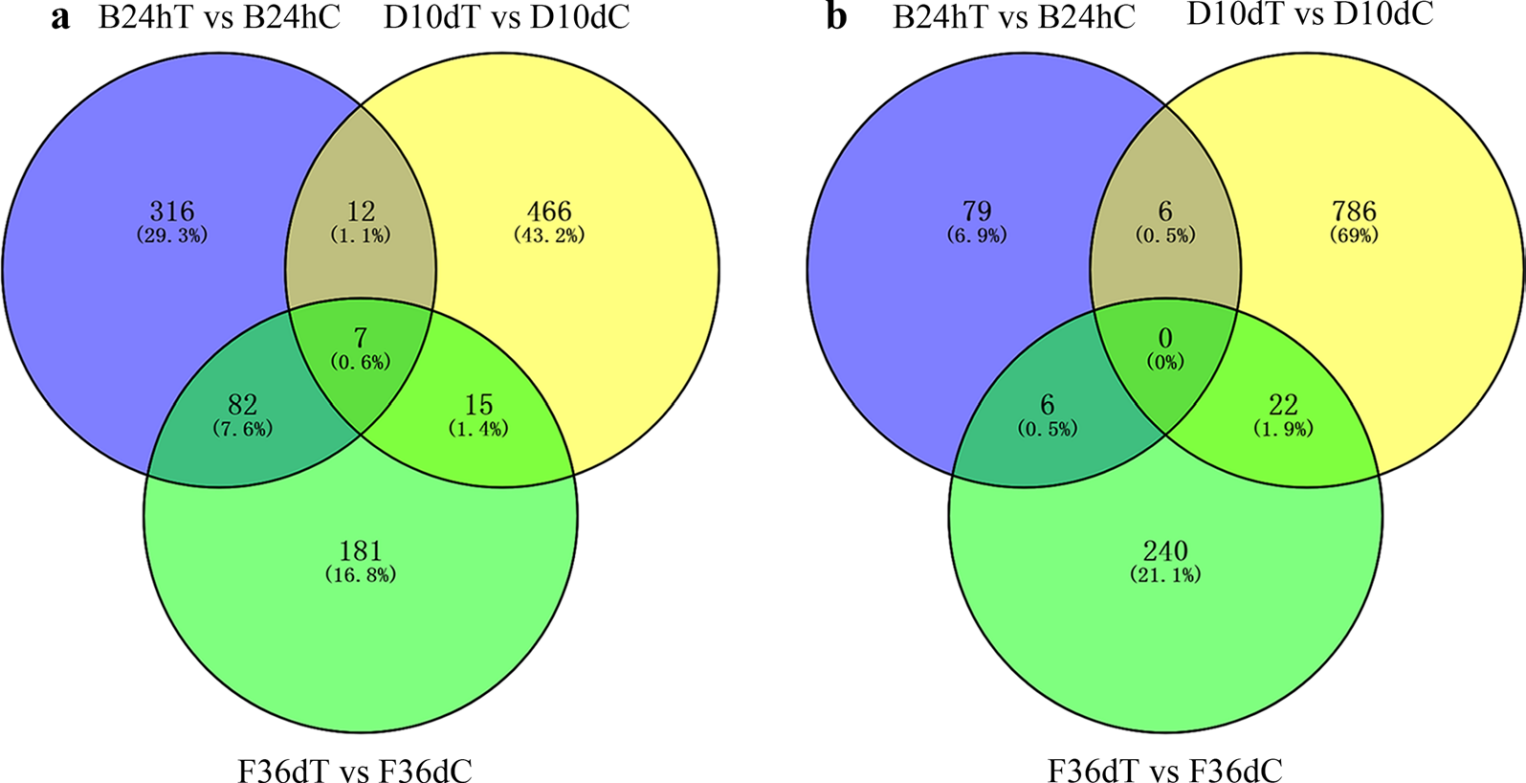
Venn diagrams showing the common and unique differential ions among 24 hpi, 10 dpi and 36 dpi in ESI+ mode (**a**) and ESI− mode (**b**)

As shown in Table 1, the serum metabolomic alterations were clear between infected groups and control groups. The number of upregulated ions was markedly greater than that of downregulated ions at 24 hpi. At 12 hpi, 59 and 81 differential ions were identified in ESI+ and ESI− mode, respectively; at 24 hpi, 417 and 91 differential ions were identified in ESI+ and ESI− mode, respectively; at 10 dpi, 500 and 814 differential ions were identified in ESI+ and ESI− mode, respectively; and at 36 dpi, 285 and 268 differential ions were identified in ESI+ and ESI− mode, respectively. These results are similar to the trend of eosinophils and anti-*T. canis* IgG antibody, whereby the difference reached a maximum at 10 dpi. All identified ions were clustered according to the expression data at different infection stages in the ESI+ and ESI− mode, respectively (Additional file 4: Figure S3), showing that the control and infection groups can be separated at different infection stages by cluster analysis. The common and unique differential ions at 24 hpi, 10 dpi and 36 dpi are shown by means of Venn diagrams (Fig. 3). Only seven common differential ions were identified in the ESI+, indicating that the host body reacts differently to different stages of *T. canis* infection.

### Identification of potential biomarkers

**Fig. 4:**
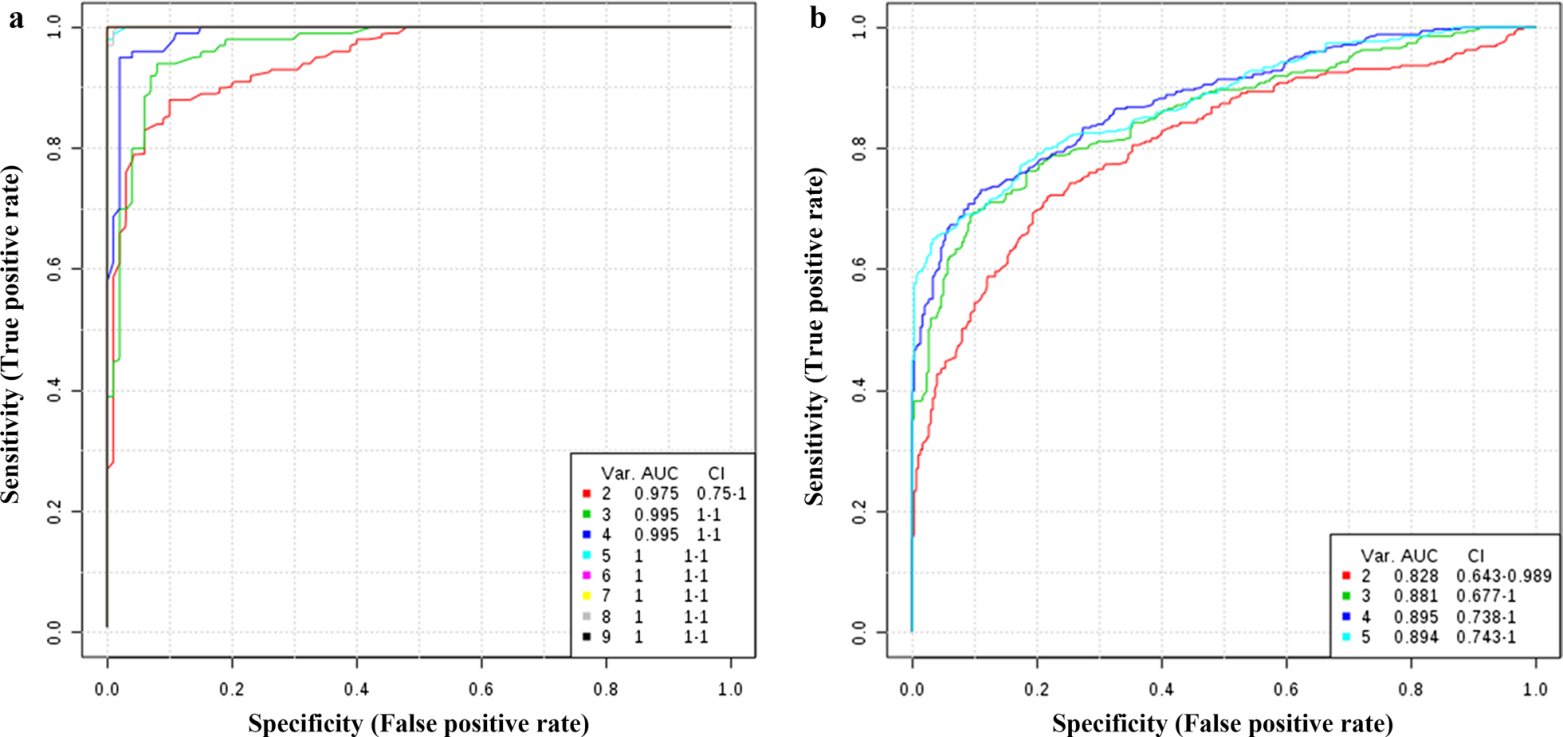
Comparisons of differential metabolites based on multivariate ROC curve analysis. **a** Biomarker metabolites identified at 12 hpi. **b** Biomarker metabolites identified at 24 hpi, 10 dpi and 36 dpi. Var. (variables) indicates the number of selected features

At 12 hpi, *T. canis* larvae were detected in the livers of 2 infected animals, but no significant difference was observed in the number of eosinophils or level of anti-*T. canis* IgG antibody between infected and control groups, and fewer differential ions were identified. Hence, 12 hpi was considered as the initial phase of *T. canis* infection, and 24 hpi, 10 dpi and 36 dpi were analyzed together as the main clinical course of *T. canis* infection. The AUC was constructed by MetaboAnalyst v.4.0 to predict the potential biomarkers. Six and three metabolites were found in ESI+ mode and ESI− mode, respectively, with AUC > 0.9, *P* < 0.05 and FC ≥ 1.2 or ≤ 0.8333 by classical univariate ROC analyses at 12 hpi. Among these, calcitroic acid was considered as an ideal biomarker with an AUC value of 1. There were three metabolites in ESI+ mode and two metabolites in ESI− mode with AUC > 0.8 by classical univariate ROC analyses at 24 hpi to 36 dpi (Table 2). Multivariate ROC curve based exploratory analysis was performed to evaluate the utility of metabolite combination for diagnosing *T. canis* infection in dogs. The AUC values were 1 (95% CI: 1–1) when using 5 potential biomarkers at 12 hpi, and 0.894 (95% CI: 0.743–1) when using all 5 potential biomarkers at 24 hpi to 36 dpi by multivariate ROC curve analysis (Fig. 4).

**Table 2:** Potential biomarker metabolites of *Toxocara canis* infection in dog serum at 12 hpi and 24 hpi to 36 dpi by LC-MS/MS

Infection stage	Ion mode	Metabolites	Entry	AUC	*P*-value	Log2(FC)	Metabolic pathways
12 hpi	ESI(+)	O-isovaleryl-l-carnitine	C20826	0.959	0.0158	− 0.4179	Not known
12 hpi	ESI(+)	TG(i-16:0/i-16:0/13:0)	HMDB0103789	0.959	0.0056	0.5935	Not known
12 hpi	ESI(+)	(2E)-3-[4-(sulfooxy)phenyl]prop-2-enoic acid	HMDB0125166	0.939	0.0057	− 1.0655	Not known
12 hpi	ESI(+)	3’-deoxydihydrostreptomycin	C03755	0.939	0.0027	0.3250	Not known
12 hpi	ESI(+)	Docosahexaenoic acid	C06429	0.918	0.0142	− 0.4043	Biosynthesis of unsaturated fatty acids
12 hpi	ESI(+)	Novapikromyin	C20740	0.918	0.0094	0.2805	Not known
12 hpi	ESI(−)	Calcitroic acid	C18230	1.000	0.0487	− 0.4470	Not known
12 hpi	ESI(−)	Docosapentaenoic acid	C16513	0.959	0.0081	− 0.4776	Biosynthesis of unsaturated fatty acids
12 hpi	ESI(−)	Monoacylglycerol	HMDB0011573	0.918	0.0086	− 0.4091	Not known
24 hpi to 36 dpi	ESI(+)	Biotin amide	C01893	0.871	2.21E^−05^	0.8046	Not known
24 hpi to 36 dpi	ESI(+)	Iridotrial	C06070	0.819	0.0067	0.3800	Biosynthesis of secondary metabolites
24 hpi to 36 dpi	ESI(+)	{[1-(4-methoxyphenyl)pentan-3-yl]oxy}sulfonic acid	HMDB0133010	0.807	0.0010	0.4903	Not known
24 hpi to 36 dpi	ESI(−)	Angiotensin (5–8)	C20972	0.810	0.0008	− 0.3484	Not known
24 hpi to 36 dpi	ESI(−)	l-Threonate	L-Threonate	0.807	0.0025	0.3041	Ascorbate and aldarate metabolism

### Metabolic pathways alterations at different infection stages

**Fig. 5:**
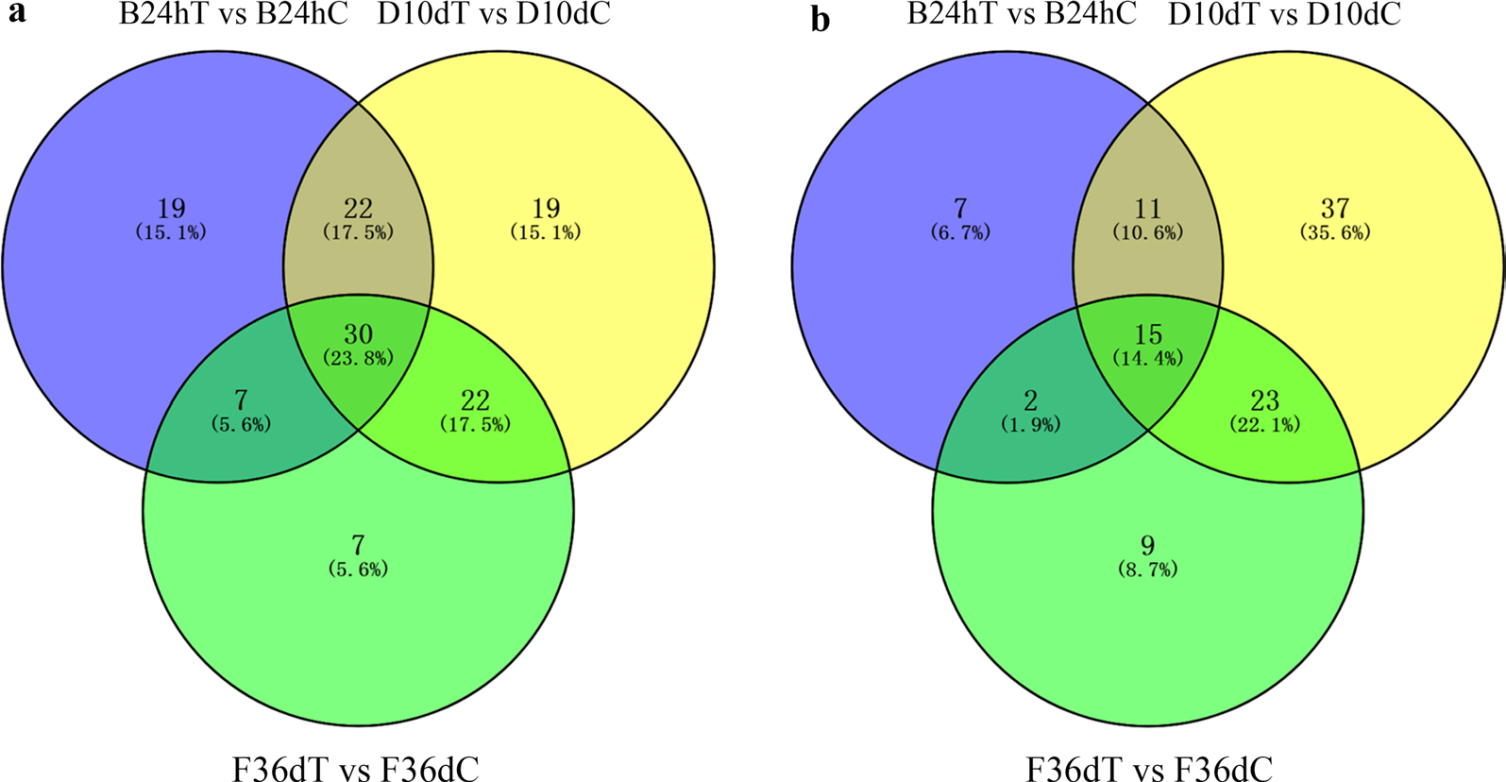
Venn diagram showing the common and unique differential metabolic pathways among 24 hpi, 10 dpi and 36 dpi in ESI+ mode (**a**) and ESI− mode (**b**)

**Fig. 6:**
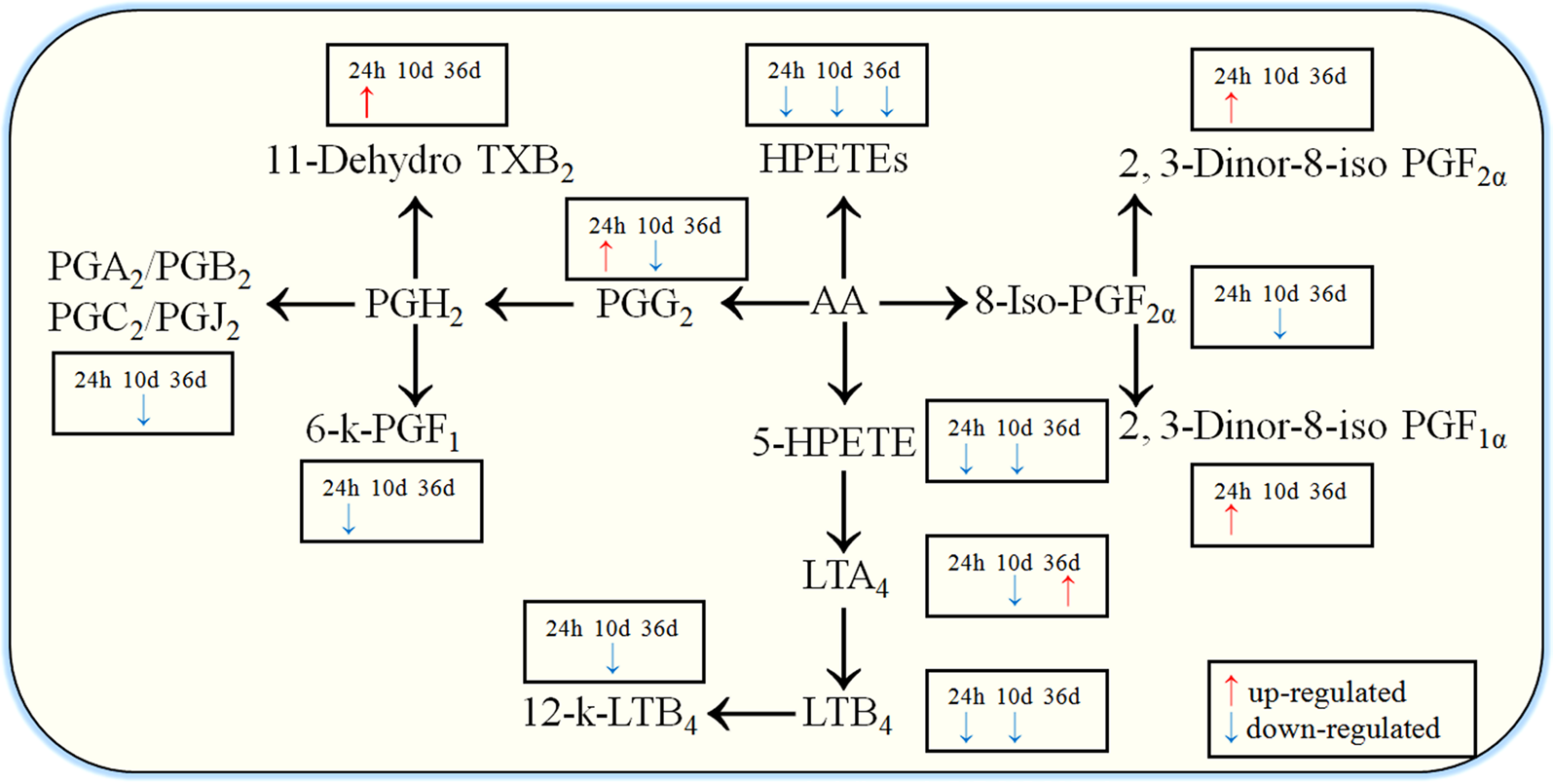
Schematic overview of the differential metabolites involved in arachidonic acid metabolites pathway at 12 hpi, 10 dpi and 36 dpi. Red and blue arrows represent upregulation and downregulation, respectively. No arrows indicate that the metabolites were not significantly different in this infection stage

A total of 1043 and 762 metabolites were identified in the ESI+ and ESI− mode, respectively, according to the KEGG and HMDB annotations. Additionally, a total of 126 and 104 differential metabolic pathways were discovered in ESI+ and ESI− mode, respectively, by the KEGG database. At 12 hpi, 41 and 42 differentially abundant metabolites were identified, which are involved potentially in 32 and 42 differential metabolic pathways in ESI+ and ESI− mode, respectively; at 24 hpi, 75 and 45 differential metabolites were identified, which are involved in 78 and 35 differential metabolic pathways in ESI+ and ESI− mode, respectively; at 10 dpi, 248 and 231 differential metabolites were identified, which are involved in differential 93 and 86 metabolic pathways in ESI+ and ESI− mode, respectively; and at 36 dpi, 109 and 47 differential metabolites were identified, which are involved in 66 and 49 differential metabolic pathways in ESI+ or ESI− mode, respectively (Additional file 5: Table S2). The differential metabolic pathways are illustrated in Fig. 5 by means of Venn diagrams in the ESI+ and ESI− mode at 24 hpi, 10 dpi and 36 dpi. Of the 126 metabolic pathways in ESI+ mode, 30 (23.8%) pathways were involved at 24 hpi, 10 dpi and 36 dpi of *T. canis* infection, and 51 (40.5%) pathways were involved in two infection stages. Of the 104 metabolic pathways in ESI− mode, 15 (14.4%) pathways were involved at 24 hpi, 10 dpi and 36 dpi of *T. canis* infection, and 36 (34.6%) pathways were involved in two infection stages. A total of 39 pathways were involved at 24 hpi, 10 dpi and 36 dpi, among which 6 pathways were involved in both ESI+ and ESI− mode. The common differential metabolic pathways at 24 hpi, 10 dpi and 36 dpi with differential metabolites ≥ 2 are listed in Additional file 6: Table S3. Some of these pathways were markedly altered, such as primary bile acid biosynthesis pathway, steroid hormone biosynthesis pathway and arachidonic acid (AA) metabolism pathway (Fig. 6).

## Discussion

In this study, a non-targeted LC-MS/MS-based serum metabolomic investigation was performed to identify *T. canis*-specific changes in metabolites and metabolic pathways, and to discover potential biomarkers of *T. canis* infection in Beagle dogs. Nine potential biomarkers were identified at 12 hpi (e.g. traumatic acid and docosapentaenoic acid) by ROC analysis, suggesting good predictive value. These compounds are mainly involved in the biosynthesis and metabolism of fatty acids. At 12 hpi to 36 dpi, five potential biomarkers were detected (e.g. iridotrial and L-threonate), which are involved in the biosynthesis of secondary metabolites, and ascorbate and aldarate metabolism. These metabolites were evaluated by multivariate ROC analysis, indicating that metabolites combination can have good diagnostic potential for detection of *T. canis* infection in dogs.

Many different metabolites were identified at 24 hpi, 10 dpi and 36 dpi; however, only seven shared differential metabolites were identified between 24 hpi, 10 dpi and 36 dpi (Fig. 3), indicating that the host body reacts differently to different stages of *T. canis* infection. Additionally, 39 potential differential metabolic pathways were identified at 24 hpi, 10 dpi and 36 dpi in dogs, indicating that some important metabolic pathways were involved in the full spectrum of *T. canis* infection in dogs.

At approximately 24 hpi, the majority of *T. canis* larvae reach the liver, the organ that plays an important role in the synthesis and secretion of bile. The primary bile acid biosynthesis pathway and bile secretion pathway were altered in dogs after *T. canis* infection. The level of cholic acid, one of the primary bile acids, was upregulated 3.28 times; many of its intermediate metabolites were also upregulated. At 10 dpi, the level of cholic acid in infected puppies was lower than that in the control group with a downregulation in the intermediate metabolites in the primary bile acid biosynthesis pathway. However, the level of taurocholate was upregulated 7.55 times and taurochenodeoxycholate was upregulated abnormally 13.82 times; both of these metabolites have anti-inflammatory activities [21, 22]. At 36 dpi, the level of chenodeoxycholate, one of the primary bile acids, was reduced to half of the normal level, as well as cholic acid. The increase of primary bile acids can increase the secretion of bile which is discharged directly into the duodenum by the liver or gall-bladder; the duodenum is also the main place where *T. canis* penetrates the intestinal wall during the first stage of infection. Therefore, a dysregulated primary bile acid biosynthesis pathway may contribute to *T. canis* migration from the duodenum to the liver during early infection. At 10 dpi, *T. canis* develops into the fourth-stage larvae after molting in the small intestine of the puppy. Whether the upregulation of taurocholate and taurochenodeoxycholate eliminates inflammation and inhibits *T. canis*, or provides a better condition for *T. canis* larval migration and development, remains to be further studied.

The steroid hormone biosynthesis pathway was significantly downregulated during the entire duration of *T. canis* infection in puppies. At 24 hpi, the level of estradiol was downregulated 0.73 times. At 10 dpi, the most significant change was the downregulation of progesterone to 0.51 times and one of its metabolites, 3α,20α,21-trihydroxy-5β-pregnan-11-one, was downregulated 0.16 times. The level of testosterone metabolites, such as dihydroandrosterone was downregulated 0.26 times. Additionally, estradiol was downregulated 0.31 times, less than that at 24 hpi. The steroid hormone biosynthesis pathway gradually restored its normal level at 36 dpi. A previous study showed that estradiol can promote *T. gondii* infection *in vitro* and *in vivo* [23]. Furthermore, it has been shown that progesterone treatment increases the parasite load of *Taenia crassiceps* in mice [24]. Sex hormones are known to affect the parasite infection course, leading to resistance or susceptibility through modulating Th1/Th2 response [25]. Therefore, it is reasonable to assume that the host immune defense system was activated *via* the downregulation of steroid hormone biosynthesis pathway during *T. canis* infection in puppies, and more intermediate metabolites in steroid hormone biosynthesis pathway may play an important role in the interaction between *T. canis* and hosts, such as estradiol and 3α,20α,21-Trihydroxy-5β-pregnan-11-one. The detailed connection between *T. canis* and other metabolites of the steroid hormone biosynthesis pathway requires further investigation.

Another notable finding was the downregulation of the biosynthesis of the unsaturated fatty acids pathway. The level of linoleic acid was downregulated 0.56 times at 24 hpi. At 10 dpi, levels of various unsaturated fatty acids, such as linoleic acid, adrenic acid, eicosapentaenoic acid and 11,14,17-eicosatrienoic acid, were downregulated to about half of the normal levels. The level of eicosapentaenoic acid was downregulated 0.83 times at 36 dpi. These results indicate that unsaturated fatty acids, which maintain the relative fluidity of cell membrane and synthesize prostaglandins (PGs), could play an important role in the *T. canis*-host interaction. Furthermore, linoleic acid metabolism pathway, α-linolenic acid metabolism pathway and AA metabolism pathway were downregulated throughout the infection stages of *T. canis* in puppies, while it is notable that the levels of 11-dehydro-thromboxane B_2_ (TXB_2_)/PGG_2_/2, 3-dinor-8-iso PGF_1α_ (which are isomers and can’t be distinguished by LC-MS/MS) were upregulated 1.5044 times and 2, 3-dinor-8-iso PGF_2α_ was upregulated 1.59 times at 24 hpi in the AA metabolism pathway (Fig. 6). However, the level of other differential metabolites involved in the AA metabolism pathway were downregulated to about half of the normal level at 24 hpi and 10 dpi. Due to the presence of many isomers in the AA metabolism pathway, it was not possible to precisely identify each differential metabolite.

Nevertheless, the present study confirmed that the levels of multiple hydroperoxyeicosatetraenoic acid (HPETEs), PGs and leukotriene were downregulated at 24 hpi and 10 dpi. The level of leukotriene A4 (LTA_4_) was upregulated 1.57 times at 36 dpi. Because nematodes cannot synthesize AA [26], they must utilize the host’s AA to synthesize and release AA metabolites which are important to their survival and virulence. A previous study showed that the level of leukotriene was upregulated in *T. canis* infection at 3, 6, 18 and 24 dpi in Wistar rats [27]. Nematode-derived signals can directly induce leukotriene production by eosinophils and that leukotriene signaling is a major contributor to nematode-induced eosinophil accumulation in the lungs [28]. However, in our study, the level of leukotriene was downregulated at 24 hpi and 10 dpi in dogs, although, the number of eosinophils was increased at the two infection stages. Therefore, we speculate that the upregulation of TXB_2_/PGG_2_/2, 3-dinor-8-iso PGF_1α/2α_ in the dogs’ serum was derived from *T. canis*, which may ensure larval migration at the initial infection stage. Subsequently, the levels of AA metabolites began to decline in the dog serum, possibly due to the slowdown of inflammatory responses of the host through downregulation of the AA metabolites pathways. This may also explain why overt clinical signs are rarely observed in dogs infected with *T. canis*.

## Conclusions

In this study, the metabolome alterations of dogs’ serum as a result of *T. canis* infection were identified, providing new insights into the mechanisms underpinning the pathophysiology of this infection. Serum metabolomic profiles were different between uninfected and infected dogs at different infection stages. Our non-targeted metabolomics study enabled the identification of defined infection stage-specific differences in metabolites and pathways involved in lipid, carbohydrate and steroid hormone metabolism. Further studies are needed to characterize the mechanisms that underpin these alterations and their potential involvement in the pathogenesis of toxocariasis, especially at the early stages of *T. canis* infection.

## Supplementary information

**Additional file 1: Table S1.** The recovery of *Toxocara canis* larvae from the liver and small intestine of puppies at 3 stages post-infection with 300 *T. canis* eggs.

**Additional file 2: Figure S1.** Principal components analysis (PCA) score scatter plots of metabolites, including control group (C), infection group (T) and quality control (QC) samples, in ESI+ (**a**) and ESI− (**b**) mode.

**Additional file 3: Figure S2.** Partial least squares discriminant analysis (PLS-DA) score scatter plots of ions at 12 hpi, 24 hpi and 36 dpi in Beagle dogs infected with 300 *T. canis* embryonated eggs. **a**–**c** PLS-DA score plots of the control group (C) and infection group (T) at 12 hpi, 24 hpi and 36 dpi in ESI+ mode. **d**–**f** PLS-DA score plots of the control group (C) and infection group (T) at 12 hpi, 24 hpi and 36 dpi in ESI− mode.

**Additional file 4: Figure S3.** Heatmaps based on hierarchical clustering of the differentially abundant ions. **a**–**d** Heatmaps of the control group (C) and infection group (T) at 12 hpi, 24 hpi, 10 dpi and 36 dpi in ESI+ mode. **e**-**h** Heatmaps of the control group (C) and infection group (T) at 12 hpi, 24 hpi, 10 dpi and 36 dpi in ESI− mode.

**Additional file 5: Table S2.** Differential pathways of differential metabolites in ESI+ and ESI− mode at different infection stages by KEGG enrichment analysis.

**Additional file 6: Table S3.** The common differential metabolic pathways with differential metabolites ≥ 2 in ESI+ or ESI− mode during *Toxocara canis* infection in Beagle dogs at 24 hpi, 10 dpi and 36 dpi.

## Data Availability

All relevant data are within the paper and its additional files. The metabolomic data are available in the MetaboLights database under Accession No. MTBLS971 (http://www.ebi.ac.uk/metabolights/MTBLS971).

## Abbreviations

LC-MS/MS: liquid chromatography–tandem mass spectrometry
ES: excretory–secretory
ROC: receiver operating characteristic
AUC: area under curve
PCA: principal components analysis
PLS-DA: partial least squares discriminant analysis
AA: arachidonic acid
PG: prostaglandins
