# Supplementary material for: Serum metabolomic alterations in Beagle dogs experimentally infected with Toxocara canis
Source: Parasit Vectors. 2019 Sep 11;12:447. doi: 10.1186/s13071-019-3703-5 (PMC6737696; doi:10.1186/s13071-019-3703-5)
Supplement: Supplementary file 1 — Additional file 1: Table S1. The recovery of Toxocara canis larvae from the liver and small intestine of puppies at 3 stages post-infection with 300 T. canis eggs. [file 13071_2019_3703_MOESM1_ESM.docx]

**Additional file 1: Table S1.** The recovery of *Toxocara canis* larvae from the liver and small intestine of puppies at 3 stages post infection with 300 *T. canis* eggs.

| Infection stage | Liver | | |  | Small intestine | | |
| --- | --- | --- | --- | --- | --- | --- | --- |
|  | No. examined | No. positive | Mean^*^ |  | No. examined | No. positive | Mean^*^ |
| 12 hpi  24 hpi  36 dpi | 7  6  6 | 2  6  2 | 0.43  6.67  0.5 |  | 7  6  6 | 0  0  6 | 0  0  47.5 |

* The average number of *T. canis* larvae recovered from each organ of the examined dogs.
