# Supplementary figures and images for: Serum metabolomic alterations in Beagle dogs experimentally infected with Toxocara canis
Source: Parasit Vectors. 2019 Sep 11;12:447. doi: 10.1186/s13071-019-3703-5 (PMC6737696; doi:10.1186/s13071-019-3703-5)

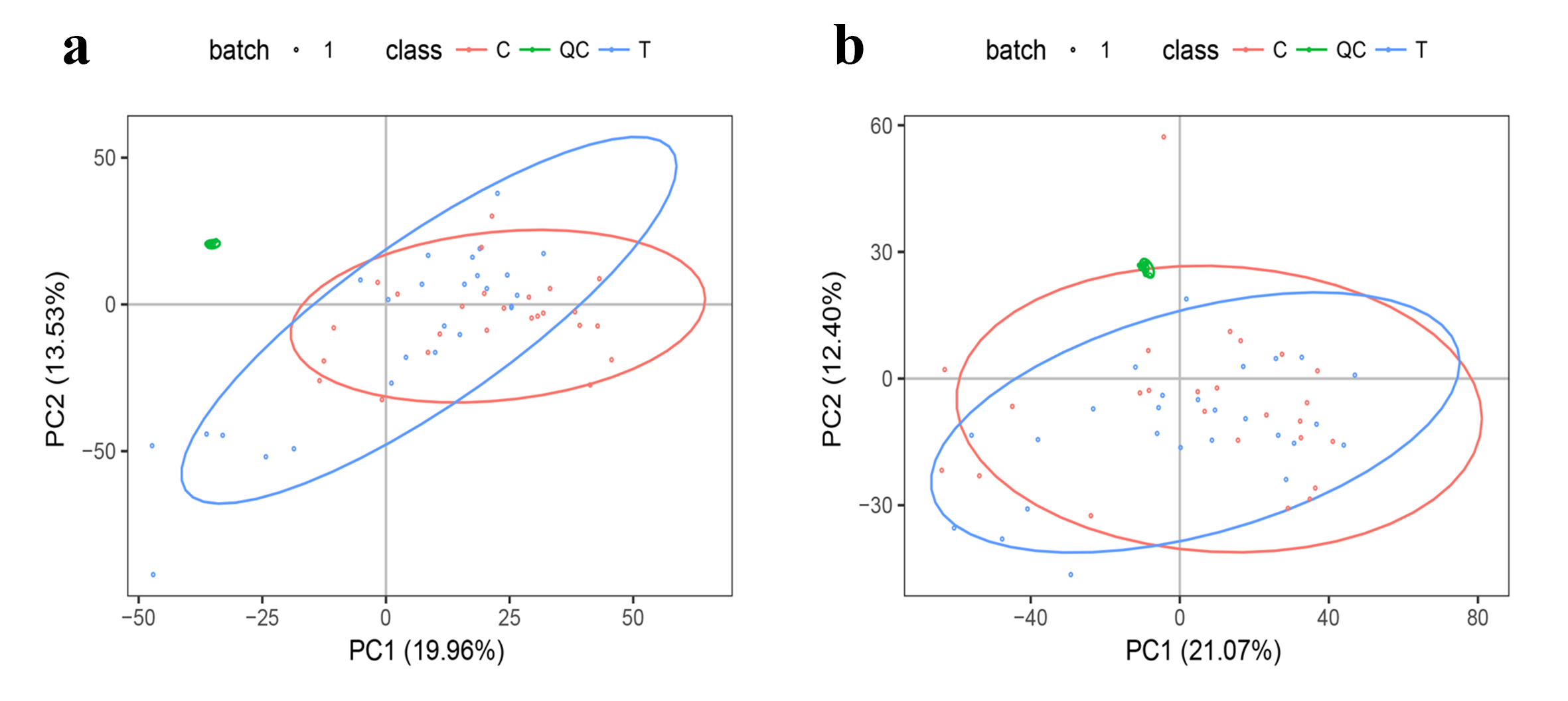

Supplement: Supplementary file 2 — Additional file 2: Figure S1. Principal components analysis (PCA) score scatter plots of metabolites, including control group (C), infection group (T) and quality control (QC) samples, in ESI+ (a) and ESI− (b) mode. [file 13071_2019_3703_MOESM2_ESM.tif]

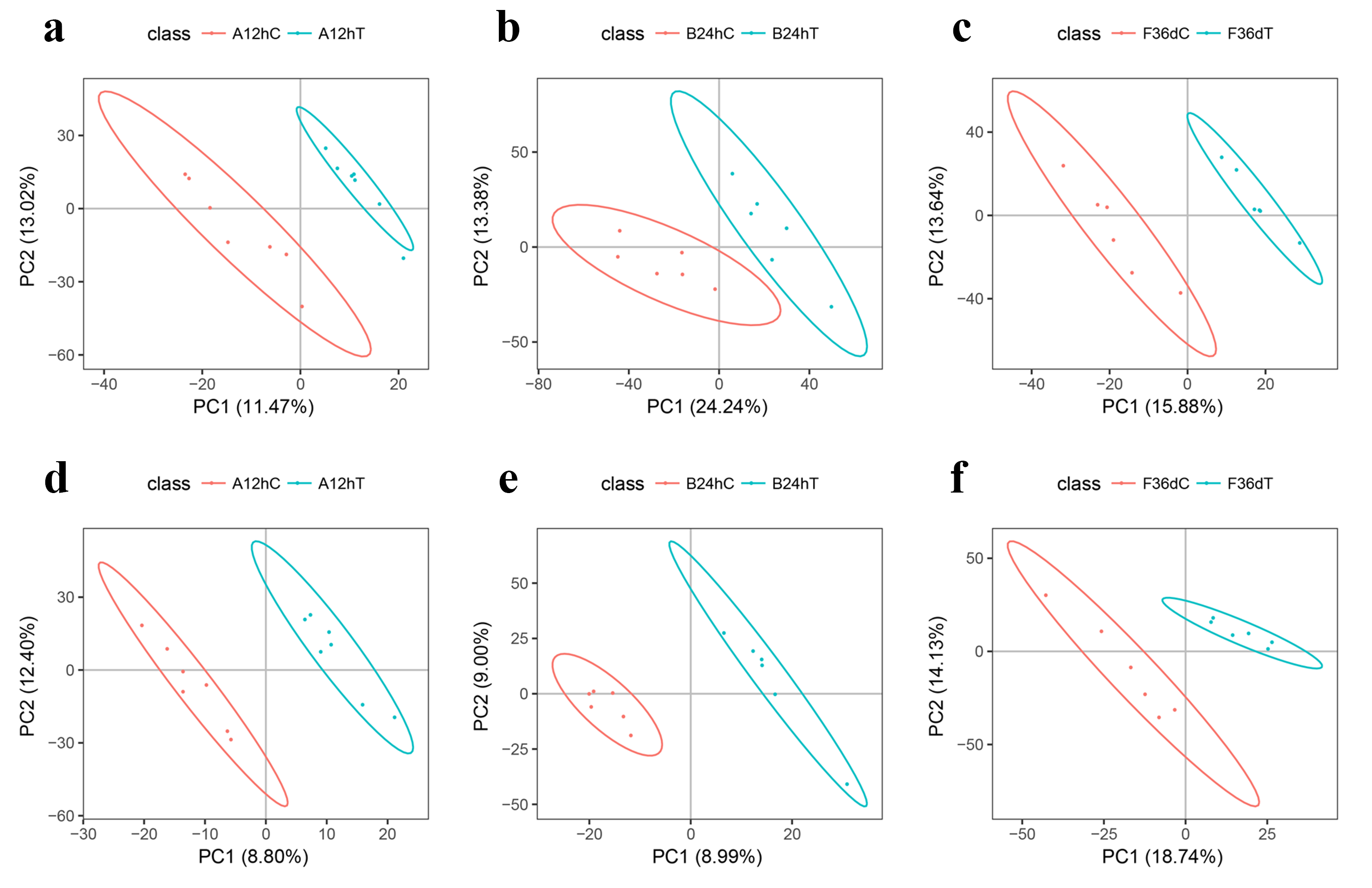

Supplement: Supplementary file 3 — Additional file 3: Figure S2. Partial least squares discriminant analysis (PLS-DA) score scatter plots of ions at 12 hpi, 24 hpi and 36 dpi in Beagle dogs infected with 300 T. canis embryonated eggs. a–c PLS-DA score plots of the control group (C) and infection group (T) at 12 hpi, 24 hpi and 36 dpi in ESI+ mode. d–f PLS-DA score plots of the control group (C) and infection group (T) at 12 hpi, 24 hpi and 36 dpi in ESI− mode. [file 13071_2019_3703_MOESM3_ESM.tif]

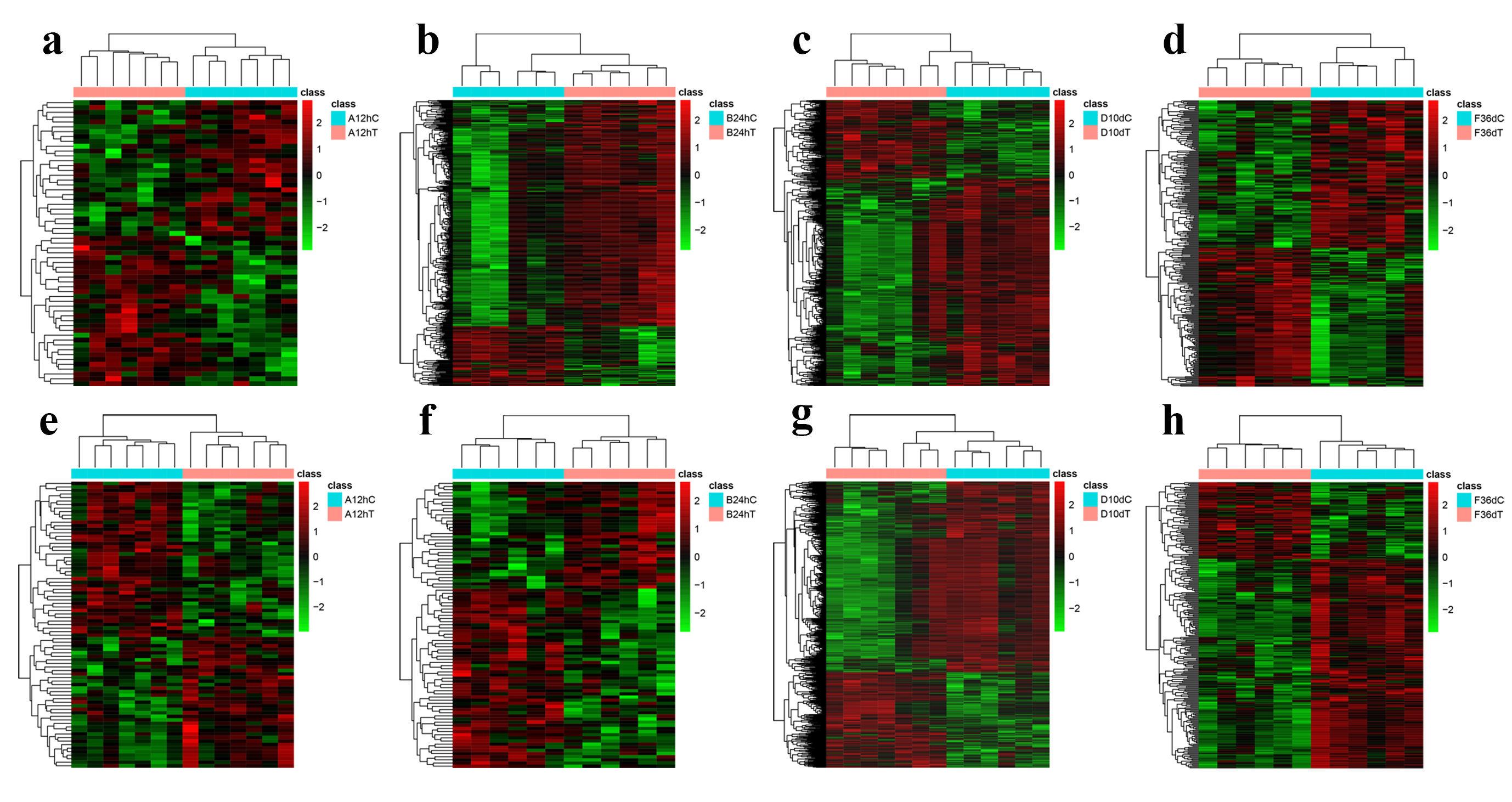

Supplement: Supplementary file 4 — Additional file 4: Figure S3. Heatmaps based on hierarchical clustering of the differentially abundant ions. a–d Heatmaps of the control group (C) and infection group (T) at 12 hpi, 24 hpi, 10 dpi and 36 dpi in ESI+ mode. e-h Heatmaps of the control group (C) and infection group (T) at 12 hpi, 24 hpi, 10 dpi and 36 dpi in ESI− mode. [file 13071_2019_3703_MOESM4_ESM.tif]
