# Supplementary material for: Serum metabolomic alterations in Beagle dogs experimentally infected with Toxocara canis
Source: Parasit Vectors. 2019 Sep 11;12:447. doi: 10.1186/s13071-019-3703-5 (PMC6737696; doi:10.1186/s13071-019-3703-5)
Supplement: Supplementary file 6 — Additional file 6: Table S3. The common differential metabolic pathways with differential metabolites ≥ 2 in ESI+ or ESI− mode during Toxocara canis infection in Beagle dogs at 24 hpi, 10 dpi and 36 dpi. [file 13071_2019_3703_MOESM6_ESM.docx]

**Additional file 6: Table S3.** The common differential metabolic pathways with differential metabolites ≥ 2 in ESI+ or ESI− mode during *Toxocara canis* infection in Beagle dogs at 24 hpi, 10 dpi and 36 dpi.

| Ion mode | Metabolic pathways | Number of enriched metabolites in a pathway | | |
| --- | --- | --- | --- | --- |
|  |  | 24 hpi | 10 dpi | 36 dpi |
| ESI(+)  ESI(+)  ESI(+)  ESI(+)  ESI(+)  ESI(+)  ESI(+)  ESI(+)  ESI(+)  ESI(+)  ESI(+)  ESI(+)  ESI(+)  ESI(+)  ESI(+)  ESI(+)  ESI(+)  ESI(+)  ESI(+)  ESI(+)  ESI(+)  ESI(+)  ESI(+)  ESI(+)  ESI(−)  ESI(−)  ESI(−)  ESI(−)  ESI(−)  ESI(−)  ESI(−)  ESI(−)  ESI(−)  ESI(−)  ESI(−)  ESI(−)  ESI(−)  ESI(−)  ESI(−) | Metabolic pathways  Steroid hormone biosynthesis  Primary bile acid biosynthesis  Serotonergic synapse  Bile secretion  2-Oxocarboxylic acid metabolism  Biosynthesis of unsaturated fatty acids  Ferroptosis  Arginine and proline metabolism  Ubiquinone and other terpenoid-quinone biosynthesis  Porphyrin and chlorophyll metabolism  Biosynthesis of amino acids  Inflammatory mediator regulation of TRP channels  Terpenoid backbone biosynthesis  Neomycin, kanamycin and gentamicin biosynthesis  Neuroactive ligand-receptor interaction  Purine metabolism  Protein digestion and absorption  Retrograde endocannabinoid signaling  Prolactin signaling pathway  Taste transduction  Histidine metabolism  Thiamine metabolism  Central carbon metabolism in cancer  Metabolic pathways  Arachidonic acid metabolism  Serotonergic synapse  Pentose and glucuronate interconversions  Bile secretion  Carbon metabolism  Galactose metabolism  Ascorbate and aldarate metabolism  Fructose and mannose metabolism  Biosynthesis of amino acids  Pentose phosphate pathway  Glucagon signaling pathway  Primary bile acid biosynthesis  Purine metabolism  Phenylalanine, tyrosine and tryptophan biosynthesis | 35^*^  9  14  2  1  1  1  7  6  5  2  3  2  4  2  2  1  1  2  1  1  1  1  1  27  15  10  1  4  5  6  3  8  2  4  4  1  1  1 | 98  35  10  18  8  4  11  1  3  3  3  2  5  1  6  4  2  2  2  3  2  2  1  1  113  24  17  16  11  4  6  7  2  7  2  1  3  1  1 | 46  3  1  1  6  9  1  3  2  3  6  5  2  4  1  2  3  2  1  1  2  2  2  2  29  4  1  2  3  5  1  2  2  2  1  1  1  2  2 |

^*^ Number of differentially abundant metabolites involved in metabolic pathways.
